# Acute Rectal Bleeding in a COVID-19 Patient: Chasing a Diagnosis

**DOI:** 10.7759/cureus.66596

**Published:** 2024-08-10

**Authors:** Hudson P Franca, Sahar S Abdelmoneim, Priscila Sole, Matthew Razavian, Odalys Frontela

**Affiliations:** 1 Internal Medicine, Larkin Community Hospital Palm Springs Campus, Hialeah, USA; 2 General Internal Medicine and Cardiovascular Medicine, Assiut University Hospital, Assiut, EGY; 3 Medicine, Centro Universitário Lusíada, São Paulo, BRA

**Keywords:** diagnosis, computed tomographic angiography, colonoscopy, hemorrhage, lower gastrointestinal bleeding

## Abstract

Guidelines for managing lower gastrointestinal bleeding (LGIB) can vary significantly, posing challenges in clinical settings. This case involves a previously healthy man who presented with severe acute rectal bleeding, along with COVID-19 positivity, Janeway lesions, and splinter hemorrhages. His condition rapidly deteriorated, with evidence suggesting a diverticular bleed. Treatment with angiography and embolization successfully stabilized him, resulting in an excellent outcome. Accurate diagnosis and stabilization necessitate a coordinated approach tailored to each patient’s condition. Early angiography should be considered for initial hemostasis in severe cases of LGIB, as demonstrated in this case.

## Introduction

Lower gastrointestinal bleeding (LGIB) presentations can be categorized as massive, moderate, or occult. Massive bleeding may appear as brisk, bright red blood (indicating a left colonic bleed) or maroon blood (indicating a right colonic bleed). Painless hematochezia is often associated with acute diverticular bleeding or angiodysplasia [1].

Diverticular bleeding may resolve spontaneously but can also present as a life-threatening condition with high mortality and a substantial risk of recurrence [2]. The diagnosis of colonic diverticular hemorrhage typically involves a colonoscopy. For intermittent bleeding, a red blood cell scan (nuclear scintigraphy) followed by single-photon emission CT may be used for localization. In unstable patients, CT angiography (CTA) is performed [2,3].

Current guidelines recommend resuscitative management to stabilize the patient and interventional imaging to identify the bleeding source. Resuscitative measures may include colonoscopy with endoscopic therapies such as submucosal epinephrine injection or endoscopic tamponade if the bleeding vessel is visible. Bipolar coagulation therapy is appropriate for non-bleeding diverticula vessels. Angiographic therapies, such as vasoconstrictor infusion and embolization, are viable alternatives when the bleeding site remains unidentified or if bleeding control via colonoscopy is unsuccessful. Surgical intervention is reserved for recurrent bleeding or after colonoscopy and angiographic interventions have failed [2].

Despite guidelines aimed at standardizing the diagnosis and management of LGIB, there is significant variability in practice. Additional comparative data may help clarify outcomes in patients receiving CTA as the primary approach versus those undergoing colonoscopy for clinically significant LGIB.

The objective of this publication is to present a case of unstable LGIB treated with CTA and embolization to achieve hemostasis and to review the literature on similar cases and their diagnostic and treatment methods.

## Case presentation

A 61-year-old male with no significant past medical history was admitted to the hospital with a two-day history of painless, massive rectal bleeding, characterized by bright red blood with clots. The patient reported generalized weakness and fever the day before presentation and had been taking two acetaminophen tablets (1,000 mg every two to three hours) for the fever over the past two days. He denied using non-steroidal anti-inflammatory drugs or aspirin. The patient had a colonoscopy 10 years prior, which showed hemorrhoids that were surgically treated with no recurrence afterward. He reported a regular healthy diet, no alcohol use, and being a nonsmoker.

**Figure 1:**
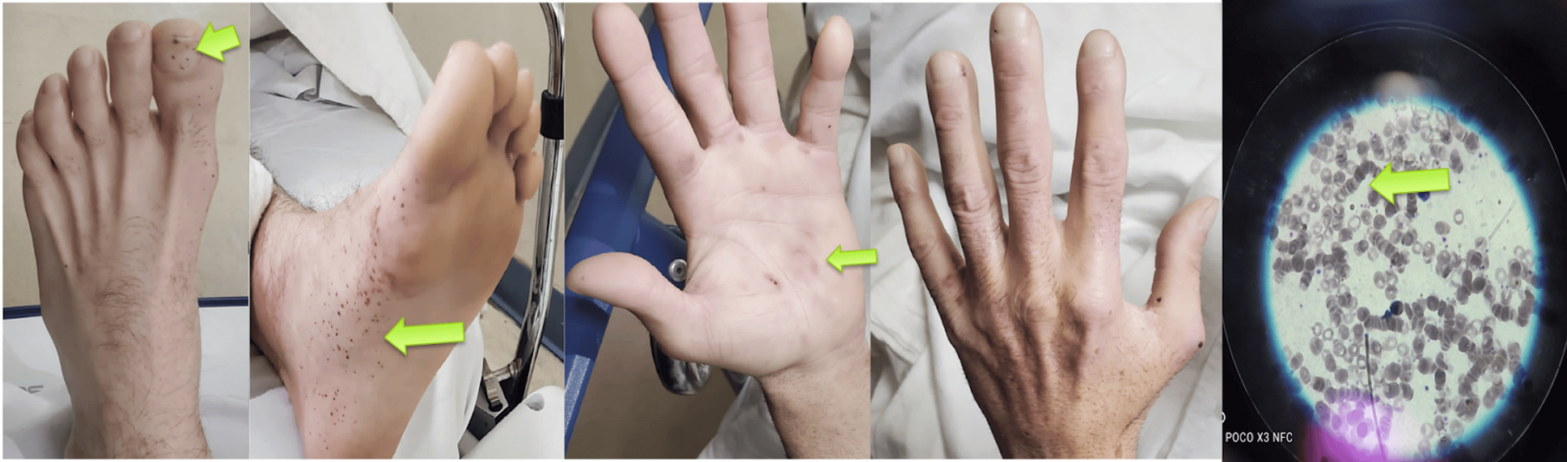
Physical examination on admission revealed splinter hemorrhages on the toes, Janeway lesions as erythematous macular lesions on the palm, and rouleaux formation on the blood smear

Upon arrival at the emergency department, the patient was tachycardic at 107 bpm, with a body temperature of 98.9 °F and blood pressure of 89/52 mmHg, indicating hemodynamic instability. The general examination revealed severe pallor, moderate dehydration, no lower extremity edema, and normal jugular venous pressure. Bilateral Janeway lesions on the hands and splinter hemorrhages on the toes were observed (Figure 1). The cardiovascular examination was normal. The abdomen was soft and non-tender, with no organomegaly. A digital rectal examination showed non-active hemorrhoids.

The patient was admitted to the ICU. Blood cultures returned negative, and baseline troponin levels were unremarkable. The complete laboratory workup (Table 1) revealed significant findings: a low hemoglobin level of 6.2 g/dL and a low hematocrit of 19.5%. Blood urea nitrogen (BUN) was within the normal range, and the BUN/creatinine ratio was 15:1, suggesting that the source of bleeding was not upper gastrointestinal. The blood smear showed rouleaux formation, characterized by the stacking of four or more red blood cells (Figure 1).

**Table 1:** Complete laboratory results The reference ranges are according to the American Board of Internal Medicine [4].

Laboratory	Serum	Reference range
Complete blood count		
Hemoglobin	6.2 g/dL	14-18 g/dL
Hematocrit	19.50%	42-50%
Mean corpuscular volume	89 fL	80-98 fL
Leucocytes	2,600/uL	4,500-11,000/uL
Neutrophils	77%	50-70%
Platelet count	189,000/mm³	150,000-450,000/uL
Reticulocyte	3.16%	0.5-1.5%
Complete metabolic panel		
Sodium	135 mEq/L	136-145 mEq/L
Potassium	4.3 mEq/L	3.5-5.0 mEq/L
Urea nitrogen	15 mg/dL	8-20 mg/dL
Creatinine	1.0 mg/dL	0.7-1.3 mg/dL
Anion gap	15.3 mmol/L	7-13 mmol/L
Calcium	7.7 mg/dL	8.6-10.2 mg/dL
Liver function tests		
Albumin	3.0 g/dL	3.5-5.5 g/dl
Total bilirubin	0.3 mg/dL	0.3-1.0 mg/dl
Alkaline phosphatase	68 U/L	30-120 U/L
Aspartate aminotransferase	56 U/L	10-40 U/L
Alanine aminotransferase	45 U/L	10-40 U/L
Coagulation studies		
Prothrombin time	11.8 seconds	11-13 seconds
Partial thromboplastin time	32 seconds	25-35 seconds
Iron studies		
Iron levels	71 mcg/dL	50-150 mcg/dL
Total iron binding capacity	176 ug/dL	250-310 ug/dL
Transferrin	135 ug/dL	200-400 mg/dL
Arterial blood gas analysis		
pH	7.3	7.38-7.44
pO2	116 mmHg	75-100 mmHg
pCO2	28 mmHg	38-42 mmHg
Bicarbonate	13.5 mEq/L	23-26 mEq/L
Immunologic studies		
Hepatitis A antibody	Negative	-
Hepatitis B surface antigen	Negative	-
Hepatitis C virus antibody	Negative	-
HIV antibody	Negative	-
COVID-19	Positive	-

**Figure 2:**
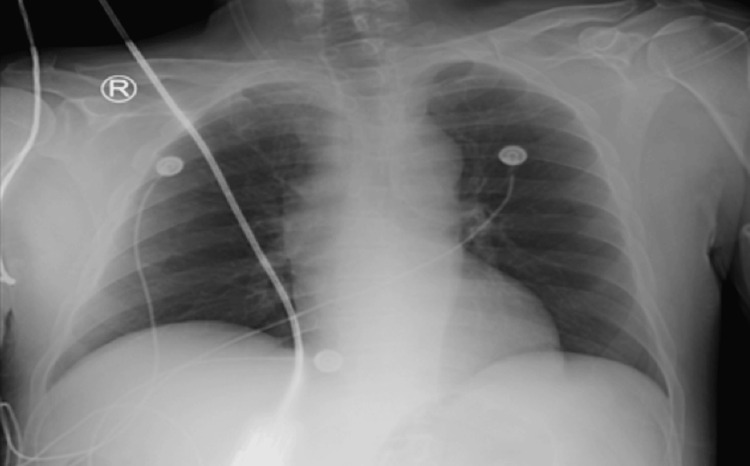
Admission chest radiograph demonstrating no abnormal findings

**Figure 3:**
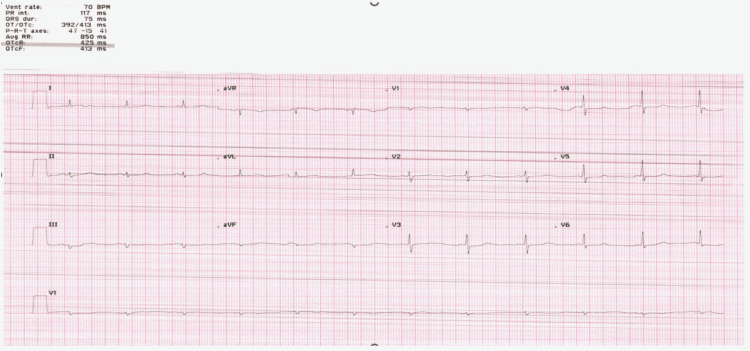
EKG showing low voltage, particularly over the limb leads, with normal sinus rhythm and left axis deviation

**Figure 4:**
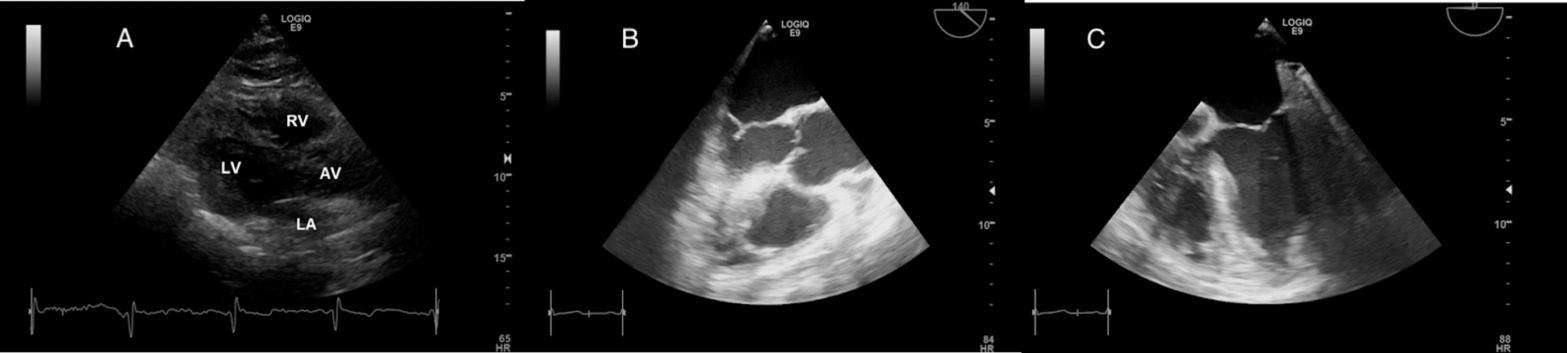
Admission echocardiography images: (A) Four-chamber view from transthoracic echocardiography showing no valve vegetation. (B) Aortic view from transesophageal echocardiography, revealing no vegetation on the aortic valve. (C) Four-chamber view at 0 degrees from transesophageal echocardiography, showing no vegetation on the mitral valve

**Figure 5:**
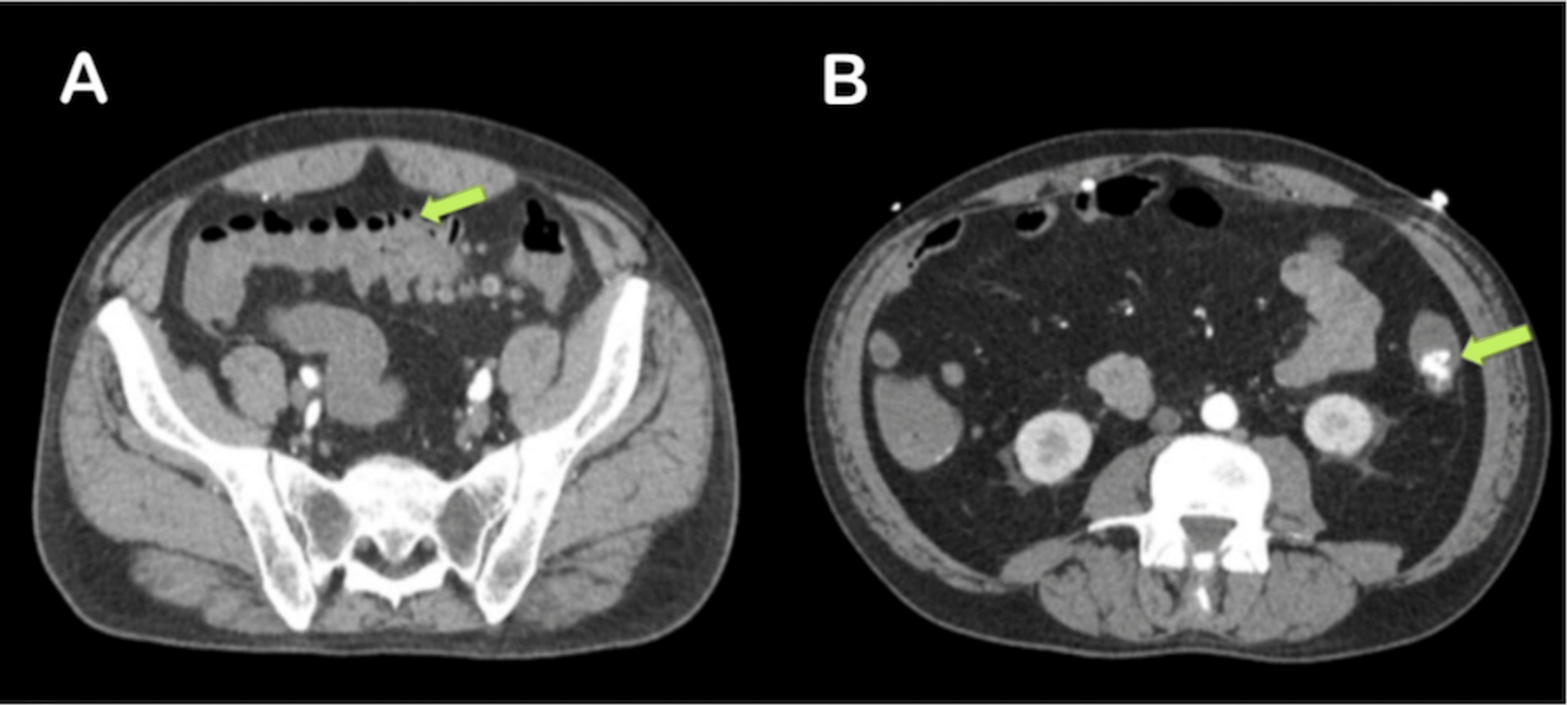
Abdominal CT with contrast demonstrating (A) diverticulosis without signs of diverticulitis and no evidence of bowel obstruction and (B) contrast extravasation within the lumen of the descending colon, indicative of a lower gastrointestinal bleed

The chest radiograph (Figure 2) was unremarkable. The ECG (Figure 3) displayed low voltage complexes, particularly over the limb leads, with a normal sinus rhythm and left axis deviation. Transthoracic echocardiography (Figure 4) revealed concentric left ventricular hypertrophy and a normal left ventricular ejection fraction of 60%. Transesophageal echocardiography showed no cardiac valve vegetation, thereby excluding the possibility of infective endocarditis (Figure 4). An emergency CT abdomen with contrast (Figure 5) identified contrast extravasation within the lumen of the descending colon, likely indicating a lower gastrointestinal bleed. The scan showed diverticulosis without evidence of diverticulitis, no bowel obstruction, and no significant peri-colonic fat stranding.

The patient was admitted to the intensive care unit, where a resuscitative protocol involving intravenous fluids and packed RBCs was initiated. The mean arterial pressure was maintained above 65 mmHg to ensure adequate cerebral perfusion. Despite these measures, the patient’s condition remained borderline, prompting the need for emergency angiography.

An angiogram, performed after selective catheterization of the inferior mesenteric artery and sub-selective catheterization of the left colic artery, revealed contrast extravasation into the descending colon. This finding was correlated with the CT scan results. Gel foam was injected into the left colic artery to achieve embolization and control the bleeding. Follow-up angiography confirmed successful embolization of the left colic artery branches, with no further contrast extravasation into the left colon.

The patient showed clinical improvement with no additional episodes of rectal bleeding, and hemoglobin levels increased to 8.9 g/dL. The patient was discharged with dietary recommendations specific to diverticulosis and was advised to follow up with a gastroenterology specialist as an outpatient.

## Discussion

The American College of Gastroenterology (ACG) guidelines recommend colonoscopy as the diagnostic test of choice for patients with LGIB, as it allows for diagnosis, mucosal tissue sampling, and potential therapeutic interventions. However, for patients with hemodynamically significant hematochezia, ACG recommends the initial use of CTA to localize the bleeding source and facilitate possible embolization [3]. Similarly, the British Society of Gastroenterology guidelines suggest CTA as the first-line investigation in unstable gastrointestinal bleeding, defined as a shock index greater than 1 [5]. A 2022 retrospective cohort study by the American Gastroenterological Association found that initial colonoscopy provided better outcomes compared to early CTA in undifferentiated LGIB, although CTA was more effective for hemostatic intervention in diverticular cases [6]. Additionally, a 2015 cohort study demonstrated a 15% increase in detecting vascular lesions in LGIB when CTA was performed before colonoscopy [7].

We present a case of a 61-year-old man, otherwise healthy and with no significant past medical history, who experienced acute rectal bleeding and hemodynamic instability within a few hours. The patient was also COVID-19 positive, which necessitates consideration of gastrointestinal pathology and inflammation factors related to the infection [8]. Research indicates that COVID-19 patients with gastrointestinal symptoms face a higher risk of complications and mortality [9]. Consequently, an urgent abdominal CT with contrast was performed, revealing extravasation within the descending colon. A multidisciplinary team identified a diverticular bleed and promptly performed an angiographic intervention. Contrast-enhanced CT proved highly effective in localizing the gastrointestinal bleeding source and facilitating CTA.

Consistent with our case, four other reports (Table 2) highlighted the effectiveness of CTA in promptly detecting the source of bleeding in hemodynamically unstable patients, thus preventing delays in clinical management.

**Table 2:** Literature review of case reports on LGIB and management using colonoscopy versus angiography (2014-2024) CTA, CT angiography; GIST, gastrointestinal stromal tumor; LGIB, lower gastrointestinal bleeding; SMA, superior mesenteric artery; TB, tuberculosis

Author	Study design	Diagnostic testing	Diagnosis	Intervention	Follow-up
Kovacs et al. (2017) [10]	Case report	(1) Endoscopy: negative; (2) Colonoscopy: negative; (3) Scintigraphy: negative; (4) CTA: positive	Meckel’s diverticulum	Laparoscopic diverticulum resection	Unremarkable recovery
Smith et al. (2021) [11]	Case report	(1) CTA: positive	Appendiceal artery hemorrhage	Embolization controlled the bleeding; laparoscopic appendectomy	Unremarkable recovery
Gonzalez-Vivo et al. (2023) [12]	Case report	(1) Endoscopy: negative; (2) Colonoscopy: negative; (3) MRI: negative; (4) CTA: positive	GIST	Laparotomy with small bowel resection	Unremarkable recovery
Bana et al. (2023) [13]	Case report	(1) Endoscopy: negative; (2) Colonoscopy: negative; (3) CT abdomen with contrast: positive; (4) CTA: positive	Abdominal TB with SMA hemorrhage	Angioembolization controlled the bleeding	The patient under TB treatment is doing well

Kovacs et al. presented a case of a young, healthy male with occasional gastrointestinal bleeding. Despite endoscopy, colonoscopy, and scintigraphy not revealing the cause, angiography eventually diagnosed Meckel's diverticulum through visualization of a vitelline artery [10].

Smith et al. described a case of a patient with large volumes of bright red blood per rectum for one day and a shock index of 0.9 who refused blood transfusions and surgery due to religious beliefs. After 18 hours of conservative management with continued bleeding, early intervention with angiography was chosen. This approach identified an appendiceal hemorrhage, which was then embolized to achieve hemostasis. Once the patient stabilized, a successful laparoscopic appendectomy was performed [11].

Gonzalez-Vivo et al. presented a case of a patient with recurrent melena who received a blood transfusion. Initial investigations, including endoscopies and colonoscopies, yielded negative results. The patient was stable and discharged. However, an MRI later revealed a pelvic mass (myoma), and despite the patient remaining stable, her hemoglobin levels continued to drop. Upon readmission, the CTA identified a gastrointestinal stromal tumor. A laparotomy was performed, followed by a small bowel resection with side-to-side anastomosis. The patient recovered and was subsequently discharged [12].

Bana et al. described a case of a patient in their mid-20s with abdominal tuberculosis who was on anti-tubercular therapy and presented with frank hematochezia. Despite receiving a blood transfusion, endoscopy, and colonoscopy did not identify the bleeding source. A CT with contrast ultimately revealed extravasation from the superior mesenteric artery, which was confirmed by angiography. Angioembolization was then performed to control the bleeding [13].

Therefore, challenges are often encountered, and patients with particularly severe ongoing bleeding who do not respond adequately to hemodynamic resuscitation or are unlikely to tolerate bowel preparation and urgent colonoscopy - like in our case - are best managed with urgent angiography. This approach not only aids in rapid diagnosis but also provides crucial information about the cause of bleeding, allowing for a more targeted and timely intervention.

## Conclusions

Our case highlighted the presentation of acute rectal bleeding in an elderly man with no significant past medical history who experienced sudden hemodynamic instability and tested positive for COVID-19 upon admission. A contrast-enhanced abdominal CT was crucial in diagnosing active gastrointestinal bleeding within the descending colon. For patients with massive, life-threatening gastrointestinal bleeding, early angiography followed by embolization proves beneficial in efficiently controlling the bleeding source, as demonstrated in our case. We emphasize the importance of a coordinated, multidisciplinary approach involving gastroenterology, interventional radiology, and surgery to effectively address the challenges associated with acute LGIB in hemodynamically unstable patients.
